# Residual deformation of coal gangue subgrade filler under multi-vibration cyclic loading

**DOI:** 10.1038/s41598-023-35199-0

**Published:** 2023-05-21

**Authors:** Xiaoyu Tang, Wen Wan, Zongtang Zhang, Wenhua Gao, Yanhao Wang

**Affiliations:** 1 School of Resources & Environment and Safety Engineering, Hunan University of Science and Technology, Xiangtan, 411201 People’s Republic of China; 2 Hunan Provincial Key Laboratory of Geotechnical Engineering for Stability Control and Health Monitoring, Hunan University of Science and Technology, Xiangtan, 411201 People’s Republic of China; 3 Hunan Software Vocational and Technical University, Xiangtan, 411201 People’s Republic of China; 4 School of Civil Engineering, Hunan University of Science and Technology, Xiangtan, 411201 People’s Republic of China

**Keywords:** Civil engineering, Environmental impact

## Abstract

In this paper, the development law of residual deformation of coal gangue subgrade filler is analyzed through large scale triaxial test, and the residual deformation model of coal gangue mainly sandstone and limestone is established. The purpose is to provide research basis for the applicability of coal gangue as subgrade filler. The results show that the deformation of coal gangue filler increases first and then tends to be constant under cyclic load of multiple vibration times. It is found that the Shenzhujiang residual deformation model cannot accurately predict the deformation law, and the corresponding modification is made to the residual deformation model of coal gangue filling body. Finally, according to the calculation of grey correlation degree, the influence degree of main factors of coal gangue filler on its residual deformation is sorted. Combined with the actual engineering situation represented by these main factors, it can be analyzed that the effect of packing particle density on residual deformation is greater than that of packing particle size composition.

## Introduction

Gangue refers to waste rock discharged during coal mine construction, coal mining, etc^1^. Excessive accumulation of coal gangue will cause serious environmental problems^2–4^. At present, the coal gangue is mainly used as concrete coarse aggregate or fine aggregate filling material, to ensure the performance of concrete under the premise of improving the recycling rate of coal gangue^5–8^. In addition, when it reaches a higher density, it has excellent engineering characteristics such as strong bearing capacity, high structural stability and less prone to excessive deformation^9^, combined with these characteristics, the use of gangue as a subgrade filler can effectively achieve the purpose of recycling. Considering that the subgrade filler will produce residual deformation under the action of circulating load^10–13^, the residual deformation will lead to safety hazards when the residual deformation is too large. Therefore, the residual deformation of gangue is one of the key points of its research on the dynamic characteristics when used as a subgrade filler.

The study of residual deformation of subgrade filler generally includes the establishment of residual deformation model and the investigation of the influencing factors of residual deformation. Residual deformation models are generally divided into two categories, namely dynamic stress-residual deformation models that describe the relationship between a certain vibration under dynamic stress and residual strain^14,15^, or vibration-residual deformation models determined directly from the residual deformation curve (such as the Shenzhujiang residual deformation model)^16–18^. Compared with the dynamic stress-residual deformation model, the vibration-residual deformation model (such as the Shenzhujiang residual deformation model) can not only describe the volume residual deformation caused by the shear characteristics of the granular materials, such as subgrade filler, but also can effectively avoid the influence of interpolation error^19^. However, the Shenzhujiang residual deformation model cannot accurately describe the residual deformation law of subgrade filler under some specific influencing factors, for example, when studying the vibration residual deformation characteristics of rockfill materials. Wang et al.^20^ found that the data fitted by Shen Zhujiang residual deformation model had greater discreteness; He et al.^21^ found that the actual lg (1 + *N*) (vibration) residual deformation curve of pile material showed obvious nonlinearity, especially under high stress conditions.

In view of the research on the influencing factors of residual deformation of the granular materials, such as subgrade filler, scholars have found that the influencing factors are diverse, including the selection of filler materials, the granule gradation of fillers, the compaction of fillers, the frequency of cyclic loads, the cyclic stress ratio, and the confining pressure of the sample^22–28^. Among them, Liu et al.^27^ pointed out that the residual deformation of the rockfill materials under the action of cyclic load is mainly due to the rearrangement of particles of the rockfill materials and the fragmentation of the edges angles of the particles of the rockfill materials, which are closely related to the compactness of the rockfill materials. In the indoor test, the confining pressure and compaction of the specimen affect its compactness. For example, scholars^29^ have also found that the confining pressure has an effect on the shear residual deformation and volume residual deformation of coarse-grained soil. At the same time, particle gradation also affects the degree of fragmentation of particles^27^. In addition, the secondary crushing of coal gangue also greatly affects the residual deformation of coal gangue^30–36^. Zheng et al.^31^ found that the main influencing factor of particle breakage of coarse-grained soil is particle shape. This particle breakage is divided into three stages, namely void filling, skeleton failure and final stability, affecting the cumulative deformation of coarse-grained soil.

In order to explore the degree of association between multivariates and residual deformation, the gray correlation method is used in this paper. The method is a statistical analysis method used to compare different factors affecting the development and change of a certain parameter^37,38^. Then it investigate the degree of correlation between different factors, which has been widely used in engineering experiments^39,40^. In the previous studies, the small triaxial or medium triaxial tests were generally used^41^. Since the grain size of coal gangue subgrade filling specimen is above 60 mm, the use of minor triaxial will not only cause the grain size of the specimen to be larger than the event size, but also cause large errors due to the size effect.

Therefore, in this paper, the experimental method is different from the small triaxial or medium triaxial tests, using large triaxial test; in terms of the number of load vibrations, different from the situation where the number of previous vibrations is small, the number of load cycles is set to 30,000 times to simulate the actual working conditions of subgrade filling. On this basis, combined with the main factors affecting the subgrade filling, this paper establishes the residual deformation model of coal gangue subgrade filling, analyzes the influence of various factors on its residual deformation. It aims to predict the settlement of coal gangue subgrade filling in practical work.

## Residual deformation test of gangue

### Specimen preparation

**Figure 1:**
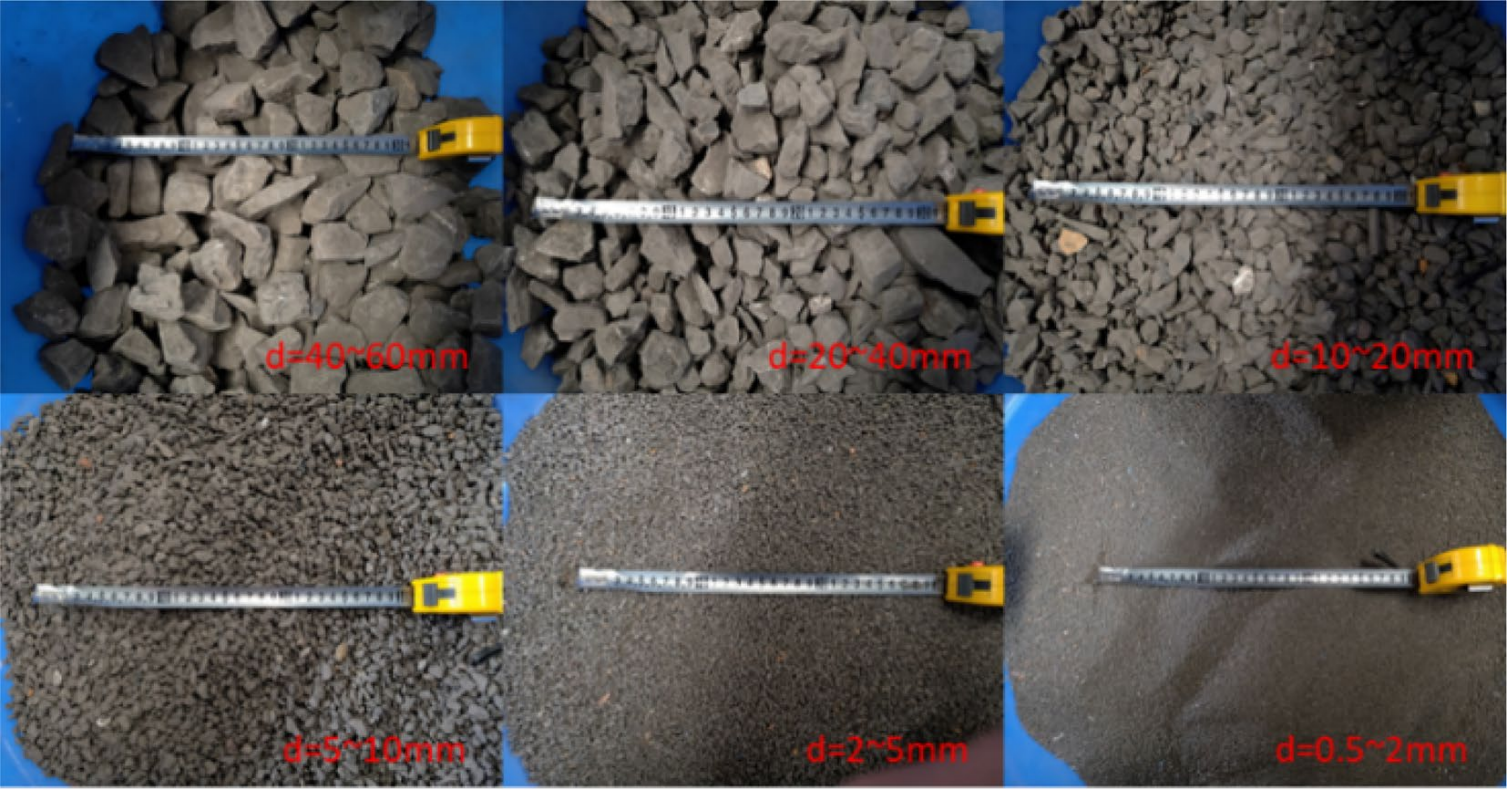
Appearance of coarse-grained filler sample.

The experimental filler sample studied in this paper, is taken from Xiangtan Tangjiashan coal mine. It is identified that the coal gangue mainly contains sandstone, limestone, shale and mudstone. The filler particle size range is large, the uniformity is poor, the particles are scattered, including more than 10 cm and less than 0.5 mm particles. Large particles have obvious edge corner, irregular shape and medium hardness. Affected by weathering, as the particle size decreases, the particles are more blurred and the texture is softer. The liquid limit of the fine-grained material of the coal gangue roadbed filler is 31.46, the plastic limit is 20.57, and the plastic index is 10.89. According to the standard (JTG D30-2015)^42^ remove the particles of more than 60 mm. The filler sample is shown in Fig. 1.

In this paper, the gradation control equation proposed by Talbot according to fractal theory is used to value the gradation parameters^43^:

1 $$d_{i} = x^{A} = \left( {{l \mathord{\left/ {\vphantom {l {l_{\max } }}} \right. \kern-0pt} {l_{\max } }}} \right){}^{A}$$

**Figure 2:**
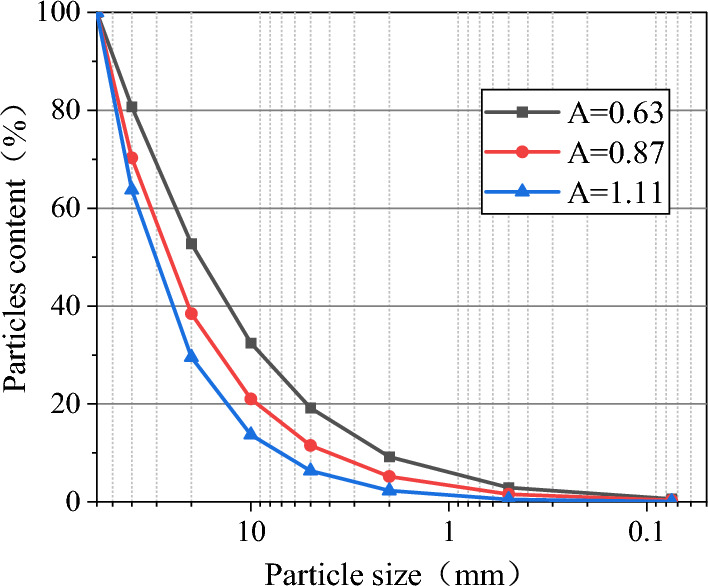
Particle gradation distribution.

Among this, *d*_*i*_ is the cumulative percentage content of particles smaller than the mass l of particle size in the specimen; *i* is the percentage of the total mass of particles with a particle size of less than one size; *l*_max_ is the maximum particle size, this test takes 60.0 mm, *A* is the graded parameter. This test selects three gradations, A = 0.63,0.87,1.11. Considering that the inhomogeneity coefficient *C*_u_ = *d*_60_/*d*_10_ > 5 and the curvature coefficient *C*_c_ = *d*_30_^2^/(*d*_60_*·d*_10_) = 1–3 are good graded, the A value is 0.3691–1.1133. The particle gradation distribution is shown in Fig. 2.

**Figure 3:**
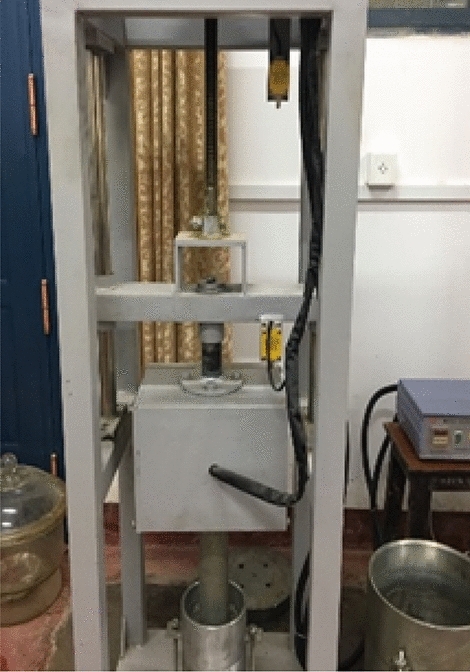
The surface vibration compaction test.

Before the large three-axis test, it is necessary to determine the density of the subgrade filler body under the maximum pressure compaction according to the standard (JTG D30-2015), through the surface vibration compaction test, as shown in Fig. 3. Then the packing sample quality is determined according to the sample volume. The surface vibration compaction test was carried out with samples of different grades. The densities of the three groups of specimens under maximum compaction were shown in Table 1.

**Table 1:** Maximum dry density of sample.

Gradation parameters A	A = 0.63	A = 0.87	A = 1.11
Maximum dry density (g/cm^3^)	1.938	1.913	1.827

**Figure 4:**
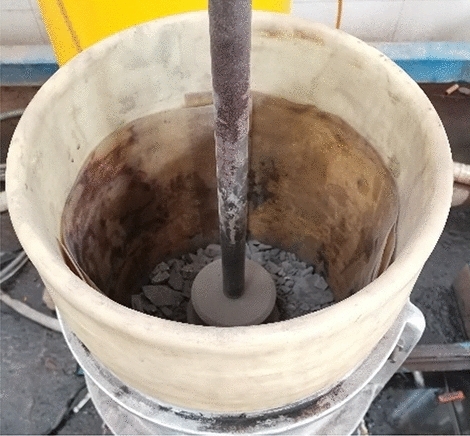
Compacted sample.

**Figure 5:**
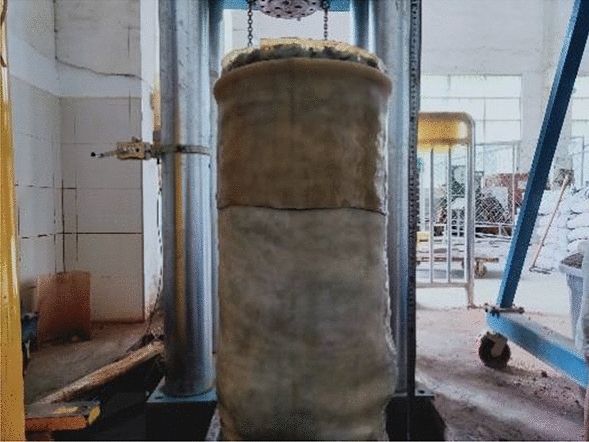
The specimen.

According to the Code for the Design of Highway Subgrades (JTG D30-2015), the test was loaded with 3–5 layers of compaction to simulate the filling process of subgrade packing in actual engineering. In the test, the compaction hammer is divided into 5 layers of compacted samples in this test, as shown in Fig. 4. The diameter of the sample is 300 mm and the height of the sample is 600 mm, as shown in Fig. 5.

### Large-scale triaxial test equipment

**Figure 6:**
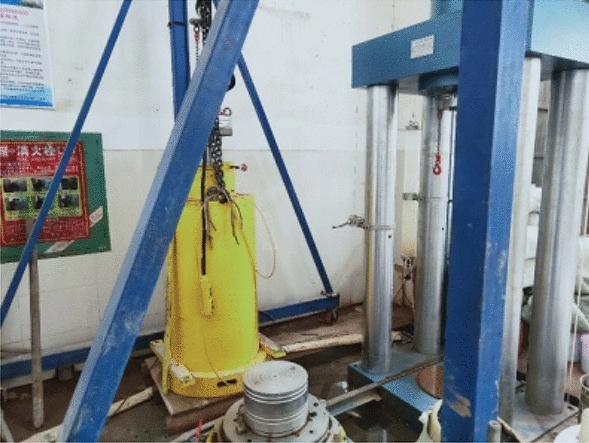
DJSZ-150 dynamic and static triaxial test system.

This study adopts the DJSZ-150 dynamic and static triaxial test system developed by Chengdu Donghua Excellence Technology Co., Ltd. and the West China Geotechnical Instrument Research Institute of Sichuan University, as shown in Fig. 6. The system is mainly designed for sample samples with large particle sizes such as gravel, coarse-grained soil and subgrade filler, which can be used to explore the dynamic and static responses of crushed stone, coarse-grained soil and subgrade filler. The system applies static load to the specimen according to displacement and load control, applies circulating load according to vibration frequency, waveform, amplitude and vibration order. The system collects axial displacement and volume deformation data through displacement meter and body change tube. In addition, the system can collect pore pressure, confining pressure and other data.

### Large-scale triaxial test scheme

According to the standard (JTG D30-2015), the compaction degree of roadbed filler must reach 93%. Therefore, the compaction levels used in this test are 90%, 93% and 95%, simulating under compaction, standard compaction and over compaction, respectively. The isobaric consolidation is used to consolidate the subgrade filler of the specimen. The cyclic load is based on the ratio of the general vehicle speed and length, and the load frequency f is 1 Hz, and the load waveform is a semi-sine wave^44^. The specimen belongs to the subgrade filler of the gravel type with good gradation, which belongs to the partial drainage material in the actual project. So, it is tested under drainage conditions. The basic test parameters in this article are shown in Table 2.

**Table 2:** Test parameters of dynamic characteristics.

Test parameters	The parameter value
Specimen size	Φ300 × 600
Consolidation stress ratio	1.0
Cyclic load waveforms	Semi-sine wave
Circulating load amplitude (kPa)	200 kPa single-level loading
Cyclic load frequency (Hz)	1.0
Number of cycle loads	30,000 times
Drainage conditions	Drain

Considering the long-term nature of the traffic load action, when simulating the traffic load, the number of cyclic load vibrations is determined to be 30,000 times, a single stage of loading. One sample was selected to control the confining pressure of 50 kPa, a compaction degree of 93%, and a grading parameter A = 0.63. The specimen number was AY01. On this basis, the three influencing factors of confining pressure, compaction degree and gradation parameters were changed. At the same time, the other two groups of influencing factors were fixed for comparative test. Among them, AP01 and AP02 changed the confining pressure size, AC01 and AC02 changed the compaction size, and AG01 and AG02 changed the particle gradation, as shown in Table 3.

**Table 3:** Accumulated deformation test plan.

Specimen number	Containment pressure (kPa)	Compaction (%)	Gradation parameters A	Circulating load form
AY01	50	93	0.63	1. Single-stage loading 2. The circulating load amplitude takes 200 kPa 3. Number of load vibrations is 30,000
AC01	100	93	0.63	
AC02	150	93	0.63	
AP01	50	90	0.63	
AP02	50	95	0.63	
AG01	50	93	0.87	
AG02	50	93	1.11	

## Residual deformation test results and deformation model of coal gangue

### Definition and value of residual deformation under cyclic loads

According to the residual deformation test scheme designed in this paper, due to the large amount of residual deformation test data, in order to facilitate comparison and calculation, combined with the characteristics of experimental data, the residual deformation is divided into three stages to take data points. The 1st to 1000th vibration is the rapid development stage of deformation (the first stage), at which the residual deformation is developed rapidly. Therefore, 1 point is extracted from every 20 points in the first stage. The 1000th to 5000th vibration is the deformation slowdown (the first stage), at which deformation gradually is slowed down. Therefore, considering the extraction of 1 point every 100 points in the second stage. In the range of 5000 to 30,000 vibration (the third stage), the deformation is almost flattened. Therefore, it is considered to extract 1 point every 500 points in the third stage. According to the development law of residual deformation of coal gangue subgrade filler, it can be found that the first and second stages are not suitable to characterize the long-term working state of the filler because the deformation is not stable. When the residual deformation develops to the third stage, the residual deformation is stable. Therefore, multiple vibration times are suitable for exploring the residual deformation of coal gangue when it is used as subgrade filler.

**Figure 7:**
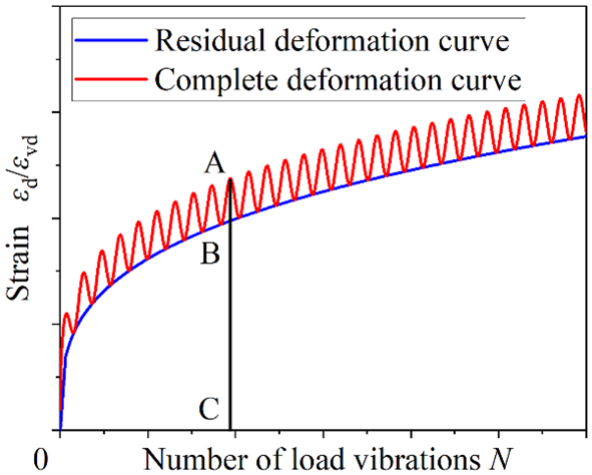
Typical magnitude–residual strain diagram.

The residual deformation is an irreparable deformation, so the residual deformation is the irreparable deformation of the specimen under cyclic load, the typical residual strain is shown in Fig. 7. The red curve is a complete deformation curve. This deformation includes recoverable deformation (such as AB line segment in Fig. 7) and unrecoverable deformation (such as BC line segment in Fig. 7), where the unrecoverable deformation is the residual deformation.

### Residual deformation under different confining pressures

**Figure 8:**
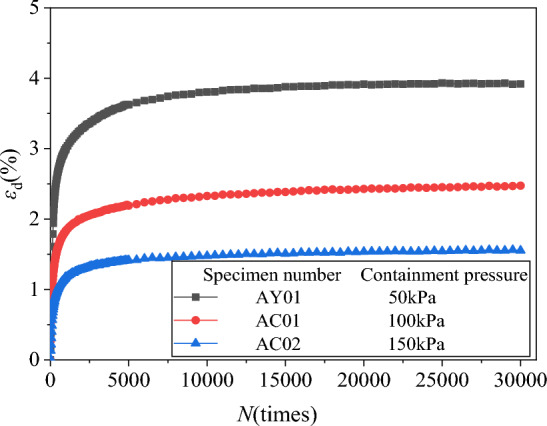
*ε*_d_-*N* relationship curves diagram under different confining pressures.

**Figure 9:**
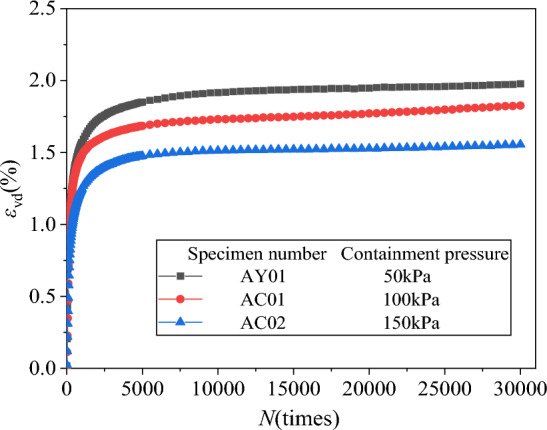
*ε*_vd_-*N* relationship curves diagram under different confining pressures.

The axial residual strain *ε*_d_ and volume residual strain *ε*_vd_ under different confining pressures were extracted. Then the axial, volumetric residual strain and vibration N relationship curves *ε*_d_-*N* and *ε*_vd_-*N* were plotted, respectively, as shown in Figs. 8 and 9.

According to Figs. 8 and 9, under the fixed circulating load amplitude, circulating load frequency and drainage conditions, etc., the cumulative strain of the specimen increases with the increase of the number of vibrations in each group of specimens, and the rate of deformation increase is first fast and then slow. The rate of deformation increase is maximized in the first 1000 times, and then gradually decreased. When the vibration is about 3000–8000 times, the curve gradually becomes flat. After 10,000 vibrations, the final curve has been flat. When the compacting degree and coarse particle content of the specimen have fixed, the residual deformation of the specimen is smaller when the surrounding pressure is larger.

The reason for this phenomenon is that as the number of vibrations increases, the subgrade filler of the specimen is gradually compacted and the stiffness gradually becomes larger, resulting in a larger growth in the initial stage of deformation, and then gradually slows down. The larger the confining pressure, the greater the compaction effect, the faster the stiffness increase, the smaller the residual deformation increment and residual deformation. In actual engineering, the confining pressure of coal gangue filler is smaller in shallow layer and larger in deep layer, so, the residual deformation of the gangue subgrade filler is larger when buried in the shallow layer. The larger the burial depth, the smaller the cumulative deformation^45^.

### Residual deformation at different degrees of compaction

**Figure 10:**
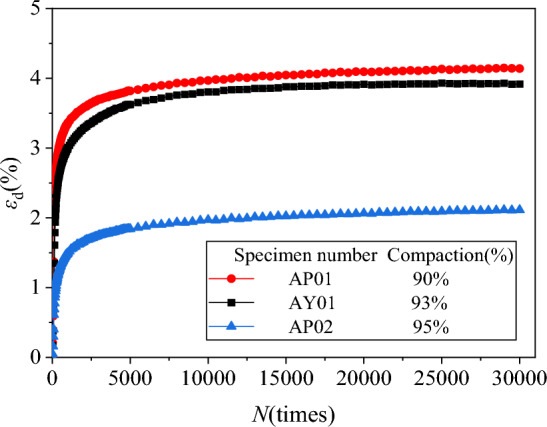
*ε*_d_-*N* relationship curves diagram under different compaction.

**Figure 11:**
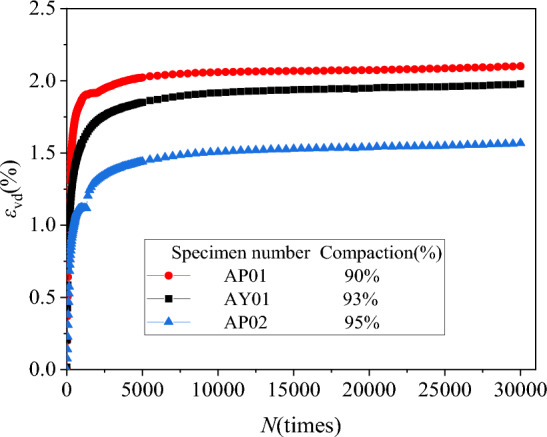
*ε*_vd_-*N* relationship curves diagram under different compaction.

Extract the axial residual strain *ε*_d_ and volume residual strain *ε*_vd_ under different degrees of compaction. We drew the axial, volumetric residual strain and vibration N relationship curves *ε*_d_-*N* and *ε*_vd_-*N*, as shown in Figs. 10 and 11.

It can be found from Figs. 10 and 11 that the basic development law of the basic *ε*_d_-*N* and* ε*_vd_-*N* relationship curve of each specimen is also the initial growth is larger, and then gradually slows down. The higher the compaction, the smaller the residual deformation of the specimen. According to the standard (JTG D30-2015), the compactness of subgrade filling needs to reach 93%. When the compactness of filling is less than 93%, the axial residual deformation will increase. The compaction degree is equal to 95%, which will significantly reduce the deformation of the gangue subgrade filler.

From Figs. 8, 9, 10 and 11, it can be found that the increase in confining pressure and the increase in compaction will reduce the final residual deformation of the specimen. This is because the increase in confining pressure and the increase in compaction have increased the compactness of the specimen, the subgrade filler compactness of the specimen is higher, and the stiffness is increased faster, thereby effectively reducing the residual deformation of the specimen.

### Residual deformation under different gradations

**Figure 12:**
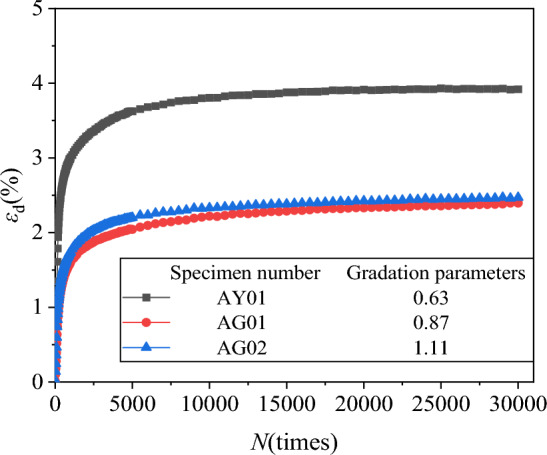
*ε*_d_-*N* relationship curves diagram under different grading.

**Figure 13:**
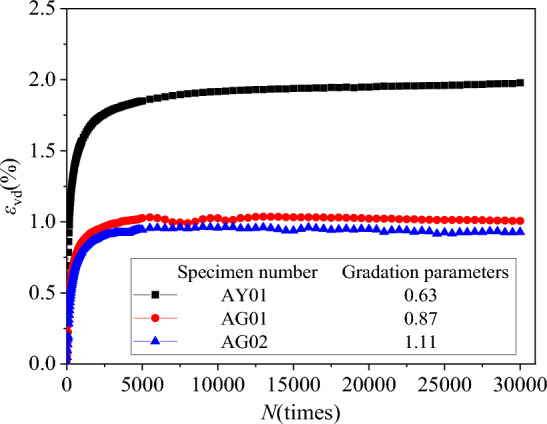
*ε*_d_-*N* relationship curves diagram under different grading.

The axial residual strain *ε*_d_ and volume residual strain *ε*_vd_ under different gradations were extracted. Then the axial, volumetric residual strain and vibration N relationship curves *ε*_d_-*N* and *ε*_vd_-*N* were plotted, as shown in Figs. 12 and 13.

It can be seen from Figs. 12 and 13 that the increase in the content of graded coarse particles will make the cumulative deformation of the specimen smaller. This is consistent with the conclusion of Qi et al.^46^. When the coarse particle content reaches a certain value, the impact on the axial residual strain is small. The reason for this phenomenon is attributed to: when there are more fine particles in the specimen, the water permeability inside the specimen will be reduced, thereby increasing the internal pore pressure at the beginning of the specimen loading, resulting in a rapid increase in the axial residual strain, while when there are more coarse particles, the pore water can be discharged in time, reducing the cumulative deformation at the beginning of the loading, thereby reducing the final axial residual deformation. In addition, during the loading process, the coarse particles will be broken twice, and the newly generated small particles will fill the particle voids, causing the increase of the overall stiffness and the decrease of the deformation.

## Gangue residual deformation model

### Shenzhujiang residual deformation model and its improved model

The Shenzhujiang residual deformation model was originally used to analyze the residual deformation of earth-rock dams and has been widely used in China. The model was carried out by Sheng^19^ on the large-scale triaxial test of the stone pile with multi-stage and small number of cycle loads. It was found that the residual deformation was proposed with the increase of the vibratile frequency in line with the semi-logarithmic law. The model takes into account the effects of confining pressure, pre-vibratile shear stress levels and vibratile frequency, and its expression is:

2 $$\varepsilon_{{{\text{vd}}}} = c_{{{\text{vd}}}} \lg \left( {1 + N} \right)$$

3 $$\gamma_{{\text{d}}} = c_{{\text{d}}} \lg \left( {1 + N} \right)$$

4 $$c_{{{\text{vd}}}} = c_{1} \gamma_{e}^{{c_{2} }} \exp \left( { - c_{3} S_{l}^{2} } \right)$$

5 $$c_{{\text{d}}} = c_{4} \gamma_{e}^{{c_{5} }} S_{l}^{2}$$

Among them, *ε*_vd_ is the volume residual strain; *γ*_d_ is shear residual strain; *N* is the time of the shock;* c*_vd_ and *c*_d_ are the slope of the volume and shear residual deformation-vibratile correlation curves, is calculated by the empirical formula of Eqs. (4) and (5);* γ*_e_ is the amplitude of the dynamic strain; *S*_*l*_ is the shear stress ratio or stress level; *c*_1_, *c*_2_, *c*_3_, *c*_4_, *c*_5_ are model parameters.

Although the Shenzhujiang residual deformation model can take into account the shear ability of the subgrade filler and obtain the relationship between the volume residual strain and the vibratile degree. However, scholars found that the follow-up stress ratio increased, and the semi-logarithmic relationship curve of the residual strain-vibratile frequency showed obvious nonlinearity^21^. Therefore, it is necessary to modify the linear relationship in the Shenzhujiang residual deformation model model into a nonlinear relationship.

### Residual deformation model of gangue filler

In the past, the Shenzhujiang residual deformation model and its improved model was mostly a multi-stage, small vibration times of dynamic experiments (within 200 vibrations). Considering the engineering background of gangue as a subgrade filler, this paper performs a large Vibration times of single-stage loading of gangue filler samples. Combined with the experimental results of “Residual deformation under different confining pressures”, “Residual deformation at different degrees of compaction” and “Residual deformation under different gradations”, the deformation curve of the gangue filler sample within 10,000 times under different conditions enters the gentle stage, that is, the residual strain value tends to be constant with the increase of the vibratile frequency, so this paper mainly analyzes the deformation model of shear residual deformation and volume residual deformation of 0–10,000 vibrations.

Since the DJSZ-150 dynamic and static triaxial test system can only collect axial displacement, it is necessary to convert the axial residual strain into shear residual strain, according to the study of ShenZhujiang's axial residual strain and shear residual strain as a linear relationship, according to formula (6) for conversion:

6 $$\gamma_{{\text{d}}} = \left( {1 + v_{d} } \right)\varepsilon_{d}$$

where, *v*_d_ is the Poisson ratio. The dynamic Poisson's ratio belongs to the characteristics of granular material, such as the Coal gangue subgrade filler, under dynamic load, which is a fixed value. In this paper, the dynamic Poisson 's ratio of coarse-grained filler is 0.33, which follows the previous research^21^.

**Figure 14:**
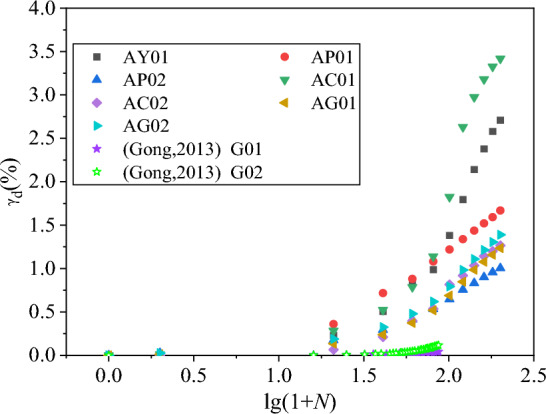
*γ*_d_-lg(1 + *N*) diagram of 0–200 vibrations.

**Figure 15:**
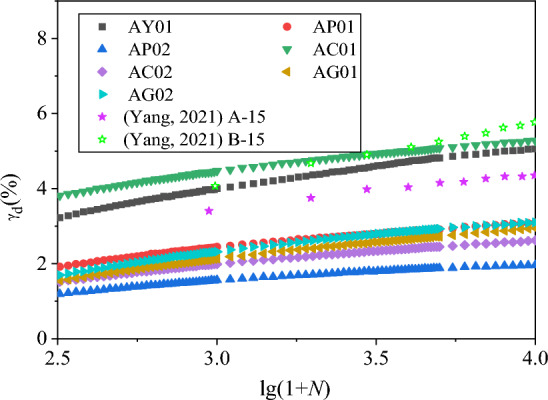
*γ*_d_-lg(1 + *N*) diagram of 201–10,000 vibrations.

**Figure 16:**
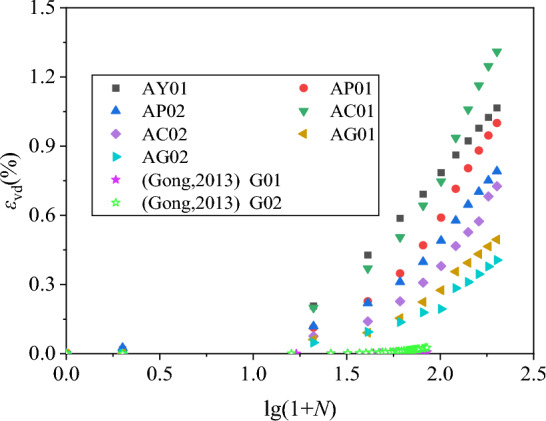
*ε*_vd_-lg(1 + *N*) diagram of 0–200 vibrations.

**Figure 17:**
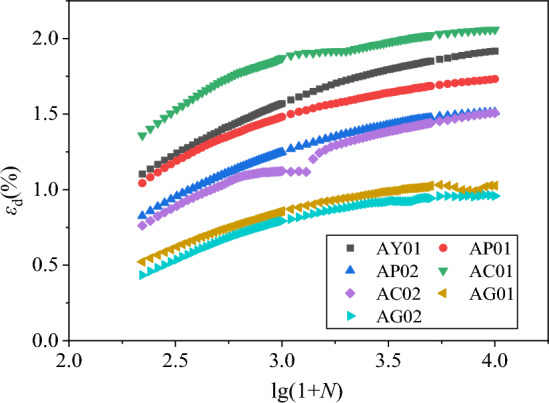
*ε*_vd_-lg(1 + *N*) diagram of 201–10,000 vibrations.

The number of vibration has been logarithmically converted. The shear residual strain-vibration pattern diagram and the volume residual strain-vibration relationship diagram are drawn separately, and they are re-divided into the residual strain-vibration pattern of 0–200 vibration times, and the residual strain-vibration correlation diagram of 201–10,000 vibration, as shown in Figs. 14, 15, 16 and 17. Considering that there are few samples in this test, we refer to the data of other studies^47,48^ and added Figs. 14, 15, 16 and 17 for comparison. Among them, the test parameter of Gong and Yang The test parameters of Gong and Yang are shown in Table 4.

**Table 4:** The test parameters of Gong and Yang.

Reference	Specimen number	Containment pressure (kPa)	Compaction (%)	Fine grain content (%)	Dynamic load amplitude
Gong et al.^47^	G01	300	–	–	180
	G02	300	–	–	540
Yang et al.^48^	A15	15	97	3	225
	B15	15	97	5	250

According to the data collation of Figs. 14, 15, 16 and 17, it can be clearly found that at 0–200 vibrations, the shear and volume residual strain show obvious nonlinearity with the increase of logarithmic vibration lg (1 + *N*). When the confining pressure is smaller, the compacting degree is lower, the coarse content is lower, the nonlinear is more obvious. This has a certain error with the Shenzhujiang residual deformation model, but it is highly matched with the improved Shenzhujiang residual deformation model such as Gong Siyi^47^. From 201 to 10,000 vibrations, the shear residual strain increased with the logarithmic vibration lg(1 + *N*), which was more in line with the prediction results of the Shenzhujiang residual deformation model. But the volume residual strain increased with the logarithmic vibration lg(1 + *N*), which had a certain nonlinearity and was in line with the logarithmic relationship.

According to the above analysis, the data are fitted so that N^*^ = lg(1 + *N*), the fitted model is shown in Table 5. The fitting parameters are shown in Table 6.

**Table 5:** Improved residual deformation model.

Residual strain	Time of shock	Morph model
Shear residual deformation	0–200	*ε*_d_ = $c_{{{\text{d}}1}}^{\prime }$·(N*)^2^
Shear residual deformation	201–10,000	*ε*_d_ = $c_{{{\text{d}}2}}^{\prime }$·N*
Volume residual deformation	0–200	*ε*_vd_ = $c_{{{\text{vd}}1}}^{\prime }$·(N*)^2^
Volume residual deformation	201–10,000	*ε*_vd_ = $c_{{{\text{vd}}2}}^{\prime }$·ln(N*)

**Table 6:** Fitting parameters of residual deformation model.

Fitting parameter	$c_{{{\text{d}}1}}^{\prime }$	$c_{{{\text{d}}2}}^{\prime }$	$c_{{{\text{vd}}1}}^{\prime }$	$c_{{{\text{vd}}2}}^{\prime }$
AY01				
Num	0.32	0.99	0.2	1.41
R^2^	0.81	0.99	0.98	0.98
AP01				
Num	0.23	0.6	0.16	1.31
R^2^	0.98	0.99	0.88	0.96
AP02				
Num	0.13	0.38	0.13	1.12
R^2^	0.91	0.99	0.9	0.96
AC01				
Num	0.41	1.08	0.22	1.61
R^2^	0.76	0.99	0.9	0.73
AC02				
Num	0.15	0.49	0.11	1.06
R^2^	0.82	0.99	0.83	0.93
AG01				
Num	0.15	0.54	0.08	0.76
R^2^	0.83	0.99	0.84	0.92
AG02				
Num	0.17	0.58	0.06	0.7
R^2^	0.86	0.99	0.85	0.88
G01				
Num	0.01	–	0.08	–
R^2^	0.93	–	0.95	–
G02				
Num	0.04	–	0.19	–
R^2^	0.88	–	0.86	–
AG01				
Num	–	–	1.07	–
R^2^	–	–	0.99	–
AG02				
Num	–	–	1.51	–
R^2^	–	–	0.99	–

According to the fitting parameters and fitting degree of the residual deformation model in Table 6, it can be found that the improved model in Table 5 can be used to describe the residual deformation characteristics of coal gangue filler under large vibration times of cycle loads, which effectively improves the fitting accuracy of the Shenzhujiang residual deformation model. At the same time, compared with other performance data, the model has better versatility.

In order to further analyze the relationship between the parameters of the model and the physical quantity of the test, the model parameters are analyzed. Under the premise of determining compaction degree and grading parameters, when confining pressure increases, ${c}_{d1}^{^{\prime}}$, ${c}_{d2}^{^{\prime}}$, ${c}_{vd1}^{^{\prime}}$ and ${c}_{vd2}^{^{\prime}}$ all decrease. When the confining pressure and grading parameters are determined, ${c}_{d1}^{^{\prime}}$, ${c}_{d2}^{^{\prime}}$, ${c}_{vd1}^{^{\prime}}$ and ${c}_{vd2}^{^{\prime}}$ all decrease with the increase of compactness. On the premise of determining the confining pressure and compaction degree, when the gradation parameter increases and the coarser content increases, ${c}_{d1}^{^{\prime}}$ and ${c}_{d2}^{^{\prime}}$ first decrease and then increase, and ${c}_{vd1}^{^{\prime}}$, ${c}_{vd2}^{^{\prime}}$ both decrease. It can be seen that confining pressure, compactness and gradation parameters can affect the parameters of the model. Considering that both Eqs. (4) and (5) in Shenzhujiang residual deformation model need to determine shear stress ratio *S*_*l*_ through static triaxial test*.* This test focuses on the residual deformation of coal gangue subgrade filling under dynamic conditions. At the same time, as a subgrade filler, considering dynamic environment is also the key research content. Therefore, the specific fitting of model parameters will be carried out in the follow-up research.

## Grey correlation analysis of residual deformation influencing factors

### Grey association analysis

The grey correlation analysis method focuses on macro analysis and basic situation between various factors. The grey correlation analysis method does not require complex and rich information^49^. The essence of this method is to analyze the degree of influence of each influencing factor on the affected object. Then identify the primary and secondary factors^50^. Therefore, the grey correlation analysis of coal gangue dynamic large-scale triaxial test is carried out to analyze the data. The correlation degree of each factor can be found out, that is, the influence degree on the deformation of gangue filler, so as to put forward guiding opinions for practical engineering.

In terms of mathematical expression, the gray association analysis method adopts the degree of influence of the gray correlation degree characterization factor on the main behavior. Its calculation method is summarized as follows^51^:

There is a system feature number column (main behavior), denoted as *Y*_0_, where:

7 $$Y_{0} = \left\{ {\left. {Y_{0} \left( l \right)} \right|l = 1,2, \ldots ,n} \right\}$$

There is also a series of systematic factors, denoted as *Y*_*i*_, *i* = 1,2,3, …, m (m is the number of factors in the series of comparisons):

8 $$Y_{i} = \left\{ {\left. {Y_{i} \left( l \right)} \right|l = 1,2, \ldots ,n} \right\}$$

Then normalize the logarithm according to the formula (9), that is, carry out dimensionless and unified data transformation on the original data:

9 $$\widehat{Y}_{i} \left( l \right) = \frac{{Y_{i} \left( l \right) - \overline{Y}_{i} }}{{\Delta Y_{i\max } }},\quad i \in \{ 0,1,2,3, \ldots ,m\}$$

wherein, $\overline{Y}_{i}$ and $\Delta Y_{i\max }$ are the maximum values of the mean and mutual difference of each series, calculated as follows Eqs. (10) and (11), respectively:

10 $$\overline{Y}_{i} = \frac{1}{l}\sum\limits_{l = 1}^{n} {Y_{i} \left( l \right)}$$

11 $$\Delta Y_{i\max } = \max \left\{ {\left. {\left| {Y_{i} \left( k \right) - Y_{i} \left( l \right)} \right|} \right|k,l = 1,2, \ldots ,n} \right\}$$

The correlation coefficient between sequence $\widehat{Y}_{0} \left( l \right)$ and series $\widehat{Y}_{i} \left( l \right)$ is *l*(1 ≦ *l* ≦ *n*)^52^:

12 $$\xi_{i} \left( l \right) = \frac{{\Delta_{\min } + \rho \Delta_{\max } }}{{\Delta_{i} l + \rho \Delta_{\max } }}$$

where *ξ*_*i*_(*l*) is the correlation coefficient of *Y*_*i*_ to *Y*_0_ in l sample;

Δ_max_ is the maximum absolute value of the difference between the total sequence and the corresponding time of the reference sequence at all moments as follows:

13 $$\Delta_{\max } = \mathop {\max }\limits_{i} \mathop {\max }\limits_{l} \left| {\widehat{Y}_{0} \left( l \right) - \widehat{Y}_{i} \left( l \right)} \right|$$

Δ_min_ is the minimum absolute value of the difference between the time of all sequences and the reference series at all moments as follows:

14 $$\Delta_{{{\text{min}}}} = \mathop {\min }\limits_{i} \mathop {\min }\limits_{l} \left| {\widehat{Y}_{0} \left( l \right) - \widehat{Y}_{i} \left( l \right)} \right|$$

Δ_*i*_(k) is the absolute value of the difference between the k-time and the reference series corresponding to the time is calculated as follows^49^:

15 $$\Delta_{i} \left( l \right) = \left| {\widehat{Y}_{0} \left( l \right) - \widehat{Y}_{i} \left( l \right)} \right|$$

*ρ* is the resolution coefficient, usually empirically based on *ρ* value, take *ρ* = 0.5^53^.

The correlation coefficient *ξ*_*i*_(*l*) only represents the degree of association between the sample data, because it has the characteristics of dispersion and not easy to compare, therefore, the introduction of the concept of correlation degree, that is, the average of the correlation coefficient, can be most of the correlation coefficients can be pooled into a value for centralized treatment. The correlation degree is calculated as follows:

16 $$r_{i} = \frac{1}{n}\sum\limits_{l} {\xi_{i} } \left( l \right)\begin{array}{*{20}c} {} & {} \\ \end{array} i = 1,2,3, \ldots {\text{n}}$$

The degree of association *r*_*i*_ represents the degree to which factor *Y*_*i*_ influences the main behavior *Y*_*0*_, for *l,j* ∈ (1, 2, 3, …n), when *Y*_*l*_ > *Y*_*k*_, then the system factor sequence *Y*_*l*_ is considered to be superior to *Y*_*k*_, or has a higher correlation with the main behavior sequence.

### Grey correlation analysis of residual strains

In order to consider the degree of correlation between the influencing factors on the residual deformation of the specimen under large vibration times, this paper sets the values of the final axial residual strain *ε*_d_ and the final volume residual strain *ε*_vd_ as the residual deformation values after 30,000 times of dynamic load loading, and set them as reference sequences, *Y*_0_ and *Z*_0_, respectively, and set the confining pressure *σ*_3_, compaction degree *η*, graded parameters *A* and vibration *N* as factor sequences, including confining pressure series *X*_1_, compaction degree series *X*_2_,and cascading parameter series *X*_3_. According to the numerical value of the factor sequence, the corresponding reference sequence is selected, and the following results are obtained through the calculation in “Grey association analysis”, Formulas (7)–(16), as shown in Table 7.

**Table 7:** All factors are related to final residual strain.

	Axial residual deformation *Y*_0_		Volume residual deformation *Z*_0_	
	Relevance	Sort	Relevance	Sort
Containment pressure *X*_1_	0.703	1	0.719	1
Compaction degree *X*_2_	0.694	2	0.712	2
Gradation parameters *X*_3_	0.681	3	0.635	3

According to the basic concept of gray correlation, it can be seen that the reference sequence representing the residual strain in this paper is positively correlated with the degree of correlation between the coefficient sequences, that is, the greater the degree of correlation, the greater the degree of influence, and vice versa.

From Table 7, it can be seen that the three influencing factors all have a certain degree of correlation with the final residual strain of gangue filler, and whether it is for axial residual strain or volume residual strain, the degree of influence is ranked by confining pressure *X*_1_, compaction *X*_2_, and gradation *X*_3_. Considering, the confining pressure and compaction degree both indicate the compactness of the gangue test piece, and the particle gradation represents the particle composition of the sample. Reflected in the actual project, considering that the material quality of gangue is general, the quality fluctuation is large, and the natural grading is difficult to achieve good grading. According to the results of the gray correlation analysis, the degree of compaction should be appropriately increased to make the filler more compact, so as to reduce the residual strain under large vibrations.

## Conclusion

In this paper, the residual deformation development law of gangue subgrade filler was explored by using a large-scale triaxial experiment, and the correlation degree between different confining pressure, compaction degree, gradation parameters and vibration and the residual deformation value of gangue subgrade filler was analyzed based on the gray correlation analysis method, and the following conclusions were obtained.

1. According to the test results, the deformation growth rate of *ε*_d_ and *ε*_vd_ of coal gangue subgrade filling gradually slows down with the increase of load vibration times. The residual deformation of coal gangue subgrade filler under different influencing factors is as follows the greater the confining pressure, the smaller the residual deformation of the sample; the higher the degree of compaction, the smaller the residual deformation of the sample; the increase of coarse grain content will make the cumulative deformation of the sample smaller.
2. The actual residual deformation of gangue filler changes nonlinearly in semi-logarithmic coordinates with the development of vibration. Define N^*^ = lg(1 + *N*). In 0–200 vibrations, (N^*^)^2^ is used instead of N^*^ to fit the relationship between shear, volume residual strain and vibration displacement, and ln(N^*^) is used instead of N^*^ to fit the relationship between volume residual strain and vibration frequency in 201–10,000 vibrations. This provides an idea for the analysis of residual deformation model of coarse-grained filler, gravel and other subgrade fillers under large cyclic loading.
3. According to the grey correlation degree between the influencing factors and the final residual deformation value of coal gangue filler, it can be found that the compactness of the sample can affect the residual deformation of the filler more than the particle composition of the sample.

## Data Availability

The datasets generated and/or analysed during the current study are not publicly available due privacy or ethical restrictions but are available from the corresponding author on reasonable request.
